# Prospective study of the association between chronotype and cardiometabolic risk among Chinese young adults

**DOI:** 10.1186/s12889-023-16902-2

**Published:** 2023-10-11

**Authors:** Tingting Li, Yang Xie, Shuman Tao, Liwei Zou, Yajuan Yang, Fangbiao Tao, Xiaoyan Wu

**Affiliations:** 1 Department of Maternal, Child and Adolescent Health, School of Public Health, Anhui Medical University, No 81 Meishan Road, Hefei, Anhui 230032 China; 2 MOE Key Laboratory of Population Health Across Life Cycle, No 81 Meishan Road, Hefei, Anhui 230032 China; 3 Anhui Provincial Key Laboratory of Population Health and Aristogenics, Anhui Medical University, No 81 Meishan Road, Hefei, Anhui 230032 China; 4 NHC Key Laboratory of Study on Abnormal Gametes and Reproductive Tract, No 81 Meishan Road, Hefei, Anhui 230032 China; 5 School of Nursing, Anhui Medical University, 15 Feicui Road, Hefei, Anhui 230601 China

**Keywords:** Chronotype, Cardiometabolic risk, Circadian rhythms, Young adults

## Abstract

**Background:**

The association of evening chronotype with cardiometabolic disease has been well established. However, the extent to which circadian rhythm disturbances independently result in risk remains unclear. This study aimed to investigate the cross-sectional and prospective longitudinal associations between chronotype and cardiometabolic risk among Chinese young adults.

**Methods:**

From April to May 2019, a total of 1 135 young adults were selected to complete the self-administered questionnaire, and 744 fasting blood samples were collected to quantify cardiometabolic parameters. From April to May 2021, 340 fasting blood samples were collected to quantify cardiometabolic parameters. The Morning and Evening Questionnaire 5 (MEQ-5) was used to assess chronotype. The cardiometabolic (CM)-risk score was the sum of standardized Z scores based on gender for the 5 indicators: waist circumference (WC), mean arterial pressure (MAP), triglyceride (TG), homeostasis model assessment for insulin resistance (HOMA-IR), and high-density lipoprotein cholesterol (HDL-C), where the HDL-C is multiplied by-1. The generalized linear model was used to determine the cross-sectional and prospective longitudinal associations between chronotype and each cardiometabolic parameter.

**Results:**

Cross-sectional association analysis showed that lower MEQ-5 scores were correlated with higher fasting insulin (*β*=-1.420, 95%*CI*: -2.386~-0.453), higher HOMA-IR (*β*=-0.301, 95%*CI*: -0.507~-0.095), and higher CM risk score (*β*=-0.063, 95%*CI*: -0.122~-0.003), even after adjustment for covariates. Prospective longitudinal association analysis also showed that lower MEQ-5 scores were associated with 2 years later higher fasting glucose (*β*=-0.018, 95%*CI*: -0.034~-0.003), higher fasting insulin (*β*=-0.384, 95%*CI*: -0.766~-0.003), higher HOMA-IR (*β*=-0.089, 95%*CI*: -0.176~-0.002), and higher CM-risk score (*β*=-0.109, 95%*CI*: -0.214~-0.003) after adjustment for covariates.

**Conclusions:**

Evening chronotype was significantly correlated with higher CM risk among young adults. Our findings suggest that biologically and socially affected sleep timing misalignment is a contributing factor to cardiovascular disease risk.

**Supplementary Information:**

The online version contains supplementary material available at 10.1186/s12889-023-16902-2.

## Background

Circadian rhythms are controlled by a master biological clock located in the suprachiasmatic nucleus of the hypothalamus, which regulates sleep and wake patterns, hormone secretion, and eating behavior [1]. One important individual difference associated with circadian rhythms is the natural variation in preferred bedtime and subjective time of peak alertness, also called the chronotype of an individual [2]. Morning chronotypes and evening chronotypes define the 2 extremes of chronotype [3]. Remarkably, biological differences between chronotypes extend beyond sleep duration, including differences in the circadian rhythm phase of body temperature, hormone secretion patterns, alertness times, and disease risk [4]. For example, evening chronotypes tend to have more health problems involving psychological [5] and neurological [6] factors, as well as higher mortality rates than morning chronotypes [7, 8]. Similarly, a study of 23 854 Finnish adults found that the evening chronotypes are associated with an increased risk of all-cause mortality [9]. Furthermore, emerging epidemiological evidence suggests that the evening chronotype is associated with cardiometabolic risk factors, such as overweight, obesity, and type 2 diabetes [10, 11]. One possible explanation is that circadian rhythm disturbances may lead to the onset of obesity, while adipose tissue can secrete large amounts of active compounds with potential effects on cardiometabolic health [12].

Cardiovascular disease is the most common cause of morbidity and mortality in the world and is a significant challenge for healthcare systems [13]. In recent years, far from decreasing, cardiometabolic risk factors that contribute to the development of cardiovascular diseases have been on the rise [14]. Currently, as a relatively novel risk factor, evening chronotype and later sleep times are associated with morbidity, involving higher rates of cardiovascular disease [8]. In addition, studies have found that evening chronotypes have a higher risk of type 2 diabetes and metabolic syndrome compared to morning chronotypes [15]. This higher metabolic risk is characteristic of the evening chronotype, which is not yet genetically driven and is primarily related to modifiable lifestyle behaviors [16]. In fact, the evening chronotype showed a lower level of physical activity, later bedtime, and later wake-up times, and they smoked and drank more compared to the morning chronotype [17]. Moreover, evening chronotypes tend to have poorer dietary habits than morning chronotypes, including eating more calories, consuming fewer fruits and vegetables, and having a higher risk of poor dietary choices, which may contribute to poor cardiovascular health [18]. It has been hypothesized that the evening chronotype may generate these behavioral and physiological risk factors due to the prolonged imbalance between the internal biological time and externally imposed work and social activity times, making them particularly susceptible to cardiovascular diseases [19].

Early adulthood is a fragile developmental stage characterized by enhanced autonomy, changes in circadian rhythm function, reward function, and emotion. This change has a direct impact on the development of sleep problems and disorders [20]. Furthermore, early adults are at higher risk for circadian rhythm disturbances and sleep problems due to delayed circadian rhythm phases, social pressures, and societal obligations [21]. At the same time, behaviors that increase metabolic risk also emerge during this period [22]. Therefore, this is an important period for identifying modifiable new risk factors and interventions to prevent circadian rhythm disturbances and metabolic disorders later in life. At present, most of the metabolic outcomes related to individual chronotypes are studied in middle-aged and elderly populations, as the adult population (> 40 years of age) has a greater metabolic risk than the younger populations (18 ~ 29 years of age) [23]. However, changes in metabolism are likely to begin before middle age, so it is necessary to conduct studies in young adults to understand the potential risk of the evening chronotype.

Based on the aforementioned evidence, it is reasonable to hypothesize that evening chronotype may be a potential risk factor for cardiometabolic disease in young adults. Thus, given the higher prevalence of evening chronotype in young adults [24], we conducted a prospective investigation of the association between chronotype and cardiometabolic risk in Chinese young adults. The primary aim of this study was to investigate the cross-sectional and prospective longitudinal associations of chronotype with cardiometabolic risk among Chinese young adults.

## Methods

### Study population

From April and May 2019, 1 179 young adults aged 15 ~ 26 were recruited from 2 universities in Anhui and Jiangxi Provinces. Among the 1 179 participants at baseline, 1 135 participants (96.3%) completed the self-administered questionnaire, and 744 (63.1%) participants attended the medical examination and provided fasting blood samples to quantify cardiometabolic parameters. After the baseline questionnaire and medical examination data were matched based on participants’ unique identification, the final data represented 729 valid cases. From April and May 2021, a follow-up survey was conducted. A total of 340 participants completed the medical examinations and provided fasting blood samples to quantify cardiometabolic parameters. After the baseline questionnaire and twice medical examination data were matched based on participants’ unique identification, the final data represented 261 valid cases. The inclusion criteria were as follows: informed consent and no cardiovascular-related disease. The exclusion criteria were as follows: participants who were physician-diagnosed had a cardiovascular-related illness and were unable to complement the assessment of the self-administered questionnaire.

The Ethics Committee of Anhui Medical University approved this study (NO: 20170291). All participants received written informed consent. All data procedures were carried out in accordance with relevant ethical guidelines and regulations associated with the declaration of Helsinki.

### Procedures

After signing the informed consent form, the participants completed the electronic questionnaire in the classroom using their smartphones, which took approximately 10 ~ 20 min. On the morning after completing the questionnaire, participants were organized to the nearest 3 A-grade hospitals for physical examination, and fasting blood samples were taken. During the two-year follow-up visit, participants completed the medical examination and provided fasting blood samples.

### Chronotype assessment

The reduced Morningness-Eveningness Questionnaire (rMEQ) was used to assess chronotype in young adults [25], and rMEQ was well-validated in China and also regarded as the MEQ-5 items (MEQ-5) [26]. The MEQ-5 contains 5 items concerning sleep-wake times, preferred time for physical and mental activities, and subjective alertness. The total score ranges from 4 to 25, where 18 to 25 points were defined as the morning chronotypes, 12 to 17 points were defined as the neutral chronotypes, and 4 to 11 points were defined as the evening chronotypes. Chronotype was examined in this study as a continuous score, with lower scores indicating greater eveningness. The Cronbach’s alpha coefficient in this study was 0.68.

### Anthropometric measurement

In this study, the participants were sent to the nearest 3 A-grade hospitals for physical examination, and their weight and height were measured by a fully automatic electronic height and weight meter (HM1000-SZ, HeMei Tech Corp., China). Participants wore light clothing, removed shoes, and stood in an anatomical position to measure height and weight. Height measurement was accurate to 0.1 cm and weight measurement was accurate to 0.1 kg.

Body mass index (BMI) was calculated as weight divided by the square of height. BMI was used to describe general obesity. According to Chinese standards, participants over 18 years of age were classified as obesity (≥ 28.0 kg/m^2^), overweight (24.0 to 27.9 kg/m^2^), normal weight (18.5 to 23.9 kg/m^2^), and underweight (< 18.5 kg/m^2^) [27]. The weight status of participants under 18 years was defined from the growth charts of Chinese children and adolescents aged 0 to 18 years, by the BMI percentiles [28]. Growth charts provide age- and sex-specific BMI cutoff values for obesity (BMI ≥ 95th percentile), overweight (85th-≤BMI < 95th percentile), normal weight (5th-≤BMI < 85th percentile), and underweight (BMI < 5th percentile) [28].

Waist circumference (WC) was measured with a soft tape measure at the narrowest point between the lowest rib and the superior border of the iliac crest during minimal respiration. The WC measurement was accurate to 0.1 cm and uses the average of 2 measurements. Waist-to-height ratio (WHtR) was calculated as WC divided by height, and central obesity was defined as WHtR at least 0.5 [29].

### Cardiometabolic risk assessment

The participants rested for a few minutes, and their resting blood pressure was measured twice from the seated position using an Omron HEM-7121 digital sphygmomanometer (Omron Healthcare, Kyoto, Japan). Mean arterial pressure (MAP) was calculated as [1/3 systolic arterial pressure + 2/3 diastolic pressure] [30]. 5 ml of fasting venous blood sample was obtained from each participant via venipuncture in the morning. Blood samples were transported to the laboratory on ice and immediately centrifuged in a refrigerated device within 30 min of collection. Biochemical parameters including triglyceride (TG), high-density lipoprotein cholesterol (HDL-C), high-sensitivity C-reactive protein (hs-CRP), total cholesterol (TC), and low-density lipoprotein cholesterol (LDL-C) were assayed on a Hitachi 7180 automatic biochemical analyzer (Hitachi, Co. Ltd., Japan). Fasting glucose concentrations were measured using glucose oxidase or hexokinase methods under strict quality control procedures. Fasting insulin was analyzed by electrochemical luminescence immunoassay on the immunoassay analyzer (Cobas e601, Roche Ltd., Switzerland). The index of insulin resistance (HOMA-IR) was calculated as [fasting insulin (µU/ml)×fasting glucose (mmol/l)]/22.5 [31]. The cardiometabolic (CM)-risk score was the sum of standardized Z scores based on gender for the 5 indicators: WC, MAP, TG, HOMA-IR, and HDL-C, where the HDL-C value is multiplied by-1 [32]. The higher the CM risk score, the higher the cardiometabolic risk [33].

### Covariates

Baseline information on age, sex (male, female), smoking (yes, no), drinking (yes, no), and socioeconomic status (low, middle, high) was obtained from an electronic questionnaire.

Cigarette use was assessed by asking “How many days did you smoke at least one cigarette per day during the past month?” With < 1 day defined as the non-cigarette-use group, and ≥ 1 day defined as the cigarette-use group.

Alcohol use was assessed by asking “How many days did you drink at least one glass of alcohol per day during the past month?” With < 1 day classified as the non-alcohol use group, and ≥ 1 day classified as the alcohol use group.

Socioeconomic status was assessed by asking “What do you think your family’s economic condition is compared with other students?” The answers are recoded into “low”, “middle” or “high”.

The physical activity of participants in the past week was assessed using the International Physical Activity Questionnaire Short Form (IPAQ-SF) [34]. IPAQ-SF includes items that assess the frequency and duration of physical activity across 3 intensity ranges: low physical activity (3.3 metabolic equivalents [MET]), moderate physical activity (4.0 MET), and high physical activity (8.0 MET). A certain intensity of physical activity level = the activity corresponding to the MET assigned value×weekly frequency (d)×daily time (min). High physical activity level was defined as meeting any one of the following criteria: 3 days or more of vigorous activity with an energy expenditure of at least 1 500 MET·min/week, or 7 days or more of any combination of activity with an energy expenditure of at least 3 000 MET·min/week. Moderate physical activity level was defined as 3 or more days of vigorous activity of at least 20 min/day, 5 or more days of moderate or low-intensity activity of at least 30 min/day, or 5 or more days of any combination of low, moderate intensity or vigorous activity with an energy expenditure of at least 600 MET·min/week. Low physical activity level was no physical activity reported or some activity reported but not enough to meet at least the moderate physical activity criteria [35].

The Pittsburgh Sleep Quality Index (PSQI) was used to assess the sleep quality of the participants in the past month [36]. 19 individual items generate 7 component scores: sleep duration, daytime dysfunction, subjective quality of sleep, medication use, sleep disorders, sleep efficiency, and sleep latency. The sum of these 7 components’ scores yields one global score, the PSQI scores, ranging from 0 ~ 21. According to the score, sleep quality can be divided into 2 types: sleep quality good (0 to 7 points) and sleep quality poor (8 to 21 points) [37].

BMI (kg/m^2^) was collected from the medical examination.

### Statistical analysis

SPSS software (version 23.0) was used for statistical analysis (SPSS, Chicago, IL, United States). First, descriptive analyses of the participants’ demographic, behavioral, adiposity, and cardiometabolic characteristics were conducted. Categorical variables are presented as frequencies (percentages), and continuous variables are presented as the mean ± standard deviation. Second, Pearson correlations between chronotype and cardiometabolic measures were calculated. Positive coefficients indicate a lower value of the specific cardiometabolic measures based on lower MEQ-5 scores, whereas negative coefficients indicate a higher value of the specific cardiometabolic measures based on lower MEQ-5 scores. Pearson correlation coefficient greater than 0.60 indicates strong correlation, 0.41 ~ 0.59 indicates moderate correlation, and less than 0.40 indicates poor correlation. Finally, the generalized linear model was used to determine the relationship of chronotype with each cardiometabolic parameter. *β* value and 95% confidence intervals (95%*CI*) were calculated for the explanatory factors and adjusted for confounding factors, including age, gender, smoking, BMI, drinking, socioeconomic status, physical activity, and sleep quality. A two-tailed *P* value < 0.05 was regarded as statistically significant.

## Results

### Descriptive statistics

The demographic and behavioral characteristics, adiposity measures, and cardiometabolic parameters of participants at baseline and two-year follow-up are shown in Table 1. At baseline, the prevalence of obesity and central obesity in young adults were 2.33% and 5.76%, respectively. At follow-up, the prevalence of obesity and central obesity in young adults was 3.06% and 11.88%, respectively.

**Table 1:** Descriptive statistics

Variables	n	Baseline	n	Two -year follow-up
Demographic and behavior characteristics				
Sex, female, %	492	67.49	145	55.56
Age (years), mean ± SD	729	19.06 ± 3.07	261	20.69 ± 0.89
Current smoker, %	47	6.45	–	–
Current drinker, %	155	21.26	–	–
Low socioeconomic status, %	156	21.40	–	–
Poor sleep quality, %	93	12.76	–	–
Low physical activity, %	119	16.32	–	–
**Adiposity measures**				
BMI (kg/m^2^), mean ± SD	729	20.76 ± 2.88	261	21.26 ± 3.24
Underweight, %	125	17.15	40	15.33
Normal weight, %	531	72.84	195	74.71
Overweight, %	56	7.68	18	6.90
Obesity, %	17	2.33	8	3.06
WC (cm), mean ± SD	729	71.05 ± 7.80	261	73.49 ± 10.21
Central obesity, %	42	5.76	31	11.88
**Cardiometabolic parameters**				
Fasting glucose (mmol/L), mean ± SD	729	4.55 ± 0.41	261	4.66 ± 0.38
HDL-C (mmol/L), mean ± SD	729	1.44 ± 0.27	261	1.58 ± 0.52
Fasting insulin (µU/mL), mean ± SD	729	54.58 ± 45.26	261	11.98 ± 9.51
TC (mmol/L), mean ± SD	729	4.08 ± 0.69	261	4.15 ± 0.70
LDL-C (mmol/L), mean ± SD	729	2.08 ± 0.50	261	2.03 ± 0.63
TG (mmol/L), mean ± SD	729	0.90 ± 0.44	261	0.89 ± 0.37
HOMA-IR, mean ± SD	729	11.12 ± 9.53	261	2.54 ± 2.17
hs-CRP, mean ± SD	729	1.12 ± 2.02	79	0.55 ± 0.52
MAP (mmHg), mean ± SD	729	85.66 ± 9.13	260	88.23 ± 8.80
CM-risk score, mean ± SD	729	0.05 ± 3.14	261	0.09 ± 2.94

### Pearson correlations between chronotype and cardiometabolic measures

**Fig. 1:**
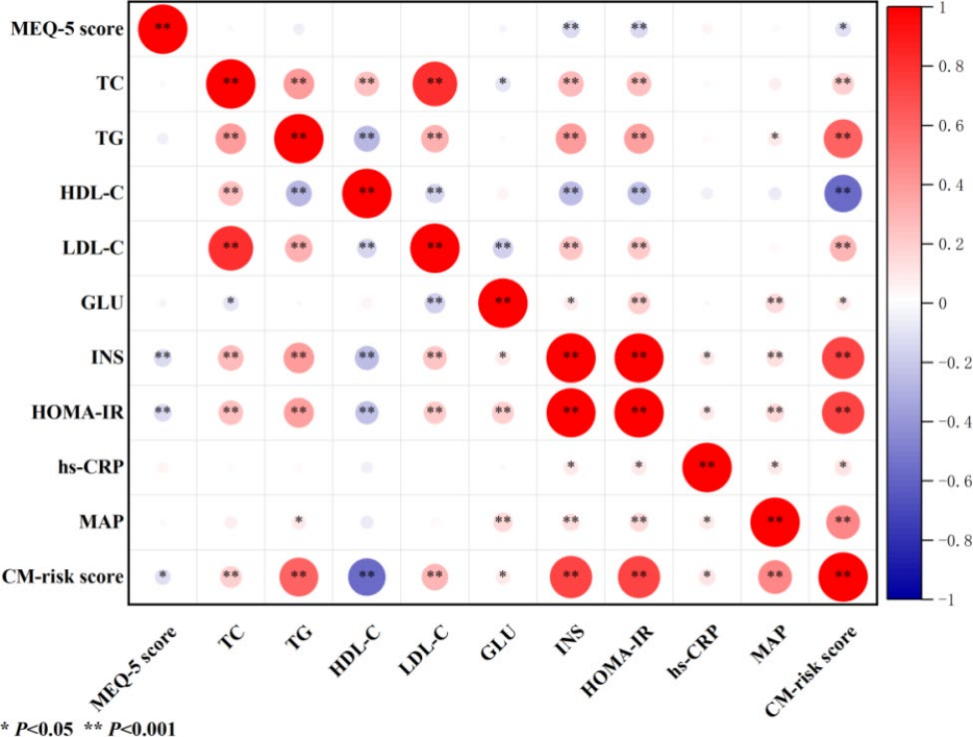
Heatmap of the association between chronotype and cardiometabolic measures. Red spots indicate a positive association and blue spots indicate a negative association

**Fig. 2:**
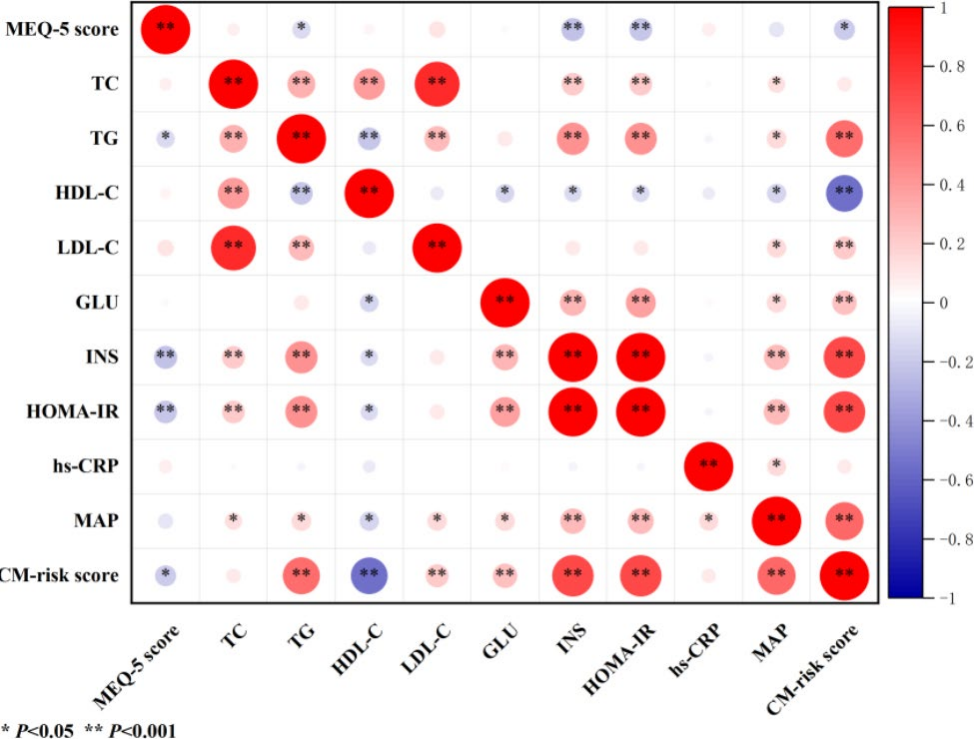
Heatmap of the association between chronotype and 2 years later cardiometabolic measures. Red spots indicate a positive association and blue spots indicate a negative association

Pearson coefficients of correlations between chronotype and cardiometabolic measures are presented in the heatmap (Fig. 1) and Table S1. The MEQ-5 score was negatively correlated with fasting insulin (*r*=-0.127, *P* = 0.001), HOMA-IR (*r*=-0.126, *P* = 0.001), and CM-risk score (*r*=-0.102, *P* = 0.006). Furthermore, Pearson coefficients of correlations between chronotype and 2-year later cardiometabolic measures are presented in the heatmap (Fig. 2) and Table S2. The MEQ-5 score was also negatively correlated with 2-year later fasting insulin (*r*=-0.161, *P* = 0.009), HOMA-IR (*r*=-0.158, *P* = 0.011), MAP (*r*=-0.135, *P* = 0.029), and CM-risk score (*r*=-0.179, *P* = 0.004).

### Cross-sectional association of chronotype and cardiometabolic parameters

The generalized linear model results showed that a lower MEQ-5 score was correlated with higher fasting insulin (*β*=-1.987, 95%*CI*: -3.115**~**-0.858), higher HOMA-IR (*β*=-0.414, 95%*CI*: -0.652**~**-0.177), and a higher CM-risk score (*β*=-0.110, 95%*CI*: -0.189**~**-0.032). After controlling for covariates, lower MEQ-5 scores still were correlated with higher fasting insulin (*β*=-1.420, 95%*CI*: -2.386~-0.453), higher HOMA-IR (*β*=-0.301, 95%*CI*: -0.507~-0.095), and higher CM-risk score (*β*=-0.063, 95%*CI*: -0.122~-0.003). As shown in Table 2.

**Table 2:** Generalized linear model analysis of chronotype and cardiometabolic parameters of young adults

Cardiometabolic parameters	Chronotype	
	Crude *β* (*95% CI*)	Adjusted *β* (*95% CI*)
TC (mmol/L)	-0.003 (-0.020 ~ 0.014)	-0.002 (-0.019 ~ 0.016)
TG (mmol/L)	-0.008 (-0.019 ~ 0.003)	-0.006 (-0.017 ~ 0.005)
HDL-C (mmol/L)	0.001 (-0.005 ~ 0.008)	0.001 (-0.006 ~ 0.007)
LDL-C (mmol/L)	0.002 (-0.011 ~ 0.014)	0.003 (-0.010 ~ 0.015)
Fasting glucose (mmol/L)	-0.003 (-0.014 ~ 0.007)	-0.006 (-0.016 ~ 0.004)
Fasting insulin (µU/mL)	-1.987 (-3.115~-0.858)^*^	-1.420 (-2.386~-0.453)^*^
HOMA-IR	-0.414 (-0.652~-0.177)^*^	-0.301 (-0.507~-0.095)^*^
hs-CRP (mg/L)	0.032 (-0.019 ~ 0.082)	0.040 (-0.011 ~ 0.092)
MAP (mmHg)	-0.041 (-0.270 ~ 0.189)	-0.011 (-0.226 ~ 0.205)
CM-risk score	-0.110 (-0.189~-0.032)^*^	-0.063 (-0.122~-0.003)^*^

Adjusted for baseline age, gender, smoking, BMI, drinking, socioeconomic status, physical activity and sleep quality

^*^P value < 0.05

### Prospective longitudinal association analysis of chronotype and 2 years later cardiometabolic parameters

The generalized linear model analysis indicated that a lower MEQ-5 score was correlated with 2 years later higher fasting insulin (*β*=-0.522, 95%*CI*: -0.911~-0.133), higher HOMA-IR (*β*=-0.117, 95%*CI*: -0.205~-0.028), higher MAP (*β*=-0.406, 95%*CI*: -0.766~-0.045), and higher CM-risk score (*β*=-0.180, 95%*CI*: -0.300~-0.060). After controlling for covariates, a lower MEQ-5 score was associated with 2 years later higher fasting glucose (*β*=-0.018, 95%*CI*: -0.034~-0.003), higher fasting insulin (*β*=-0.384, 95%*CI*: -0.766~-0.003), higher HOMA-IR (*β*=-0.089, 95%*CI*: -0.176~-0.002), and higher CM-risk score (*β*=-0.109, 95%*CI*: -0.214~-0.003). As shown in Table 3.

**Table 3:** Generalized linear model analysis of chronotype and 2 years later cardiometabolic parameters of young adults

Cardiometabolic parameters	Chronotype	
	Crude *β* (*95% CI*)	Adjusted *β* (*95% CI*)
TC (mmol/L)	0.017 (-0.011 ~ 0.046)	0.020 (-0.010 ~ 0.049)
TG (mmol/L)	-0.009 (-0.024 ~ 0.006)	-0.006 (-0.020 ~ 0.009)
HDL-C (mmol/L)	-0.000 (-0.022 ~ 0.021)	-0.006 (-0.027 ~ 0.016)
LDL-C (mmol/L)	-0.003 (-0.024 ~ 0.029)	0.010 (-0.016 ~ 0.036)
Fasting glucose (mmol/L)	-0.014 (-0.030 ~ 0.002)	-0.018 (-0.034~-0.003)^*^
Fasting insulin (µU/mL)	-0.522 (-0.911~-0.133)^*^	-0.384 (-0.766~-0.003)^*^
HOMA-IR	-0.117 (-0.205~-0.028)^*^	-0.089 (-0.176~-0.002)^*^
hs-CRP (mg/L)	-0.031 (-0.068 ~ 0.006)	-0.040 (-0.080 ~ 0.000)^*^
MAP (mmHg)	-0.406 (-0.766~-0.045)^*^	-0.281 (-0.615 ~ 0.052)
CM-risk score	-0.180 (-0.300~-0.060)^*^	-0.109 (-0.214~-0.003)^*^

Adjusted for baseline age, gender, smoking, BMI, drinking, socioeconomic status, physical activity and sleep quality

^*^P value < 0.05

## Discussion

The primary aim of this research was to investigate whether evening chronotype is correlated with cardiometabolic risk factors among Chinese young adults. Our findings suggest that evening chronotype is correlated with higher cardiometabolic risk, particularly with HOMA-IR and fasting insulin components, in both cross-sectional and prospective longitudinal associations. In addition, these effects persisted even after adjusting for covariates. To the best of our knowledge, this is the first study to show that evening chronotype is correlated with many cardiometabolic risk factors among Chinese young adults, independent of poor sleep and health behaviors.

This research indicates that the prevalence of obesity in young adults was 2.33% and 3.06% at baseline and follow-up, respectively. It was at a lower level compared with domestic and foreign studies. For instance, in a survey of 1 220 college students in Beijing, China, the rate of obesity was 3.5% [38]. Similarly, a study of 9 275 university students in Korea found that the rate of obesity was 17.8% [39]. Furthermore, in this study, the rate of central obesity in young adults was 5.76% and 11.88% at baseline and follow-up, respectively, which was at a lower level compared to other domestic and foreign studies. For example, in a survey of 4 552 university students in China, the rate of central obesity was 10.7% [40]. Likewise, a study of young adults in Bangladesh found that the rate of central obesity was 20.7% [41]. However, another study of 4 226 college students in Shandong, China found that the rate of central obesity was 5.44%, which was lower than that of our study [42].

In this study, we used a continuous CM risk score to comprehensively evaluate cardiometabolic health among young adults. In adolescents and young adults, a continuum of CM risk scores, which can detect more subtle or earlier manifestations of cardiometabolic disease, is increasingly being considered rather than a single risk factor [43]. Given this, we calculated the CM risk score for all participants and found that evening chronotype was significantly correlated with higher CM risk scores in young adults at baseline and at the two-year follow-up. Even after adjusting for covariates, evening chronotypes were still correlated with higher CM risk at baseline and at the two-year follow-up. Similar to this study, a study of Spanish middle-aged and elderly people found that evening chronotypes had a higher prevalence of metabolic syndrome than neutral chronotypes and morning chronotypes [44]. However, in another study of shift workers, chronotype was not correlated with differences in any metabolic risk factors [45].

At the same time, we also found that the evening chronotype was correlated with higher concentrations of insulin and HOMA-IR. Remarkably, HOMA-IR indices are homeostasis measures of the basal function of insulin and glucose, while postprandial dynamic measures, such as oral glucose tolerance tests, quantify the dynamic interaction of β-cell insulin secretion and insulin sensitivity [46]. There is evidence that both postprandial and fasting glucose measurements predict type 2 diabetes, which may indicate 2 different risk factors that may result in diabetes through different physiological mechanisms [47, 48]. Furthermore, postprandial hyperglycemia predicts increased all-cause mortality and cardiovascular disease, even in individuals with normal fasting blood glucose and insulin levels [49, 50]. Because postprandial glucose metabolism was not measured in this research, the effect of chronotype on glucose metabolism may be underestimated. To fully investigate whether these forms of circadian rhythm disturbances contribute to metabolic disorders and subsequent cardiovascular risk, future research should include postprandial and fasting measures.

Several explanations have been proposed for the relationship of evening chronotype with cardiometabolic disease. One explanation is that the difference in sleep duration between free days and weekdays may cause circadian dysregulation leading to chronic sleep deprivation, which is associated with health problems [51]. Another explanation is that the evening chronotype exposed to more artificial light at night can inhibit melatonin and may reduce the total amount of melatonin secreted, putting them at risk of cardiometabolic disease [52]. Recently, melatonin has been thought to play an important role in metabolism [53]. In a large cohort study, lower melatonin levels were linked to an increased risk of developing diabetes [54]. In addition, reduced melatonin levels and melatonin receptor mutations have been linked to an increased risk of type 2 diabetes [55].

Our research has several limitations. First, there were no objective measures of the circadian rhythm phase in this sample. Second, before the COVID-19 outbreak the baseline survey was conducted, and after the COVID-19 outbreak had stabilized the follow-up investigation was conducted, which may have influenced the lifestyle habits of at least some of the participants. On the other hand, our research has several strengths. For instance, we used a longitudinal design to survey the association of chronotype with CM risk over two years. Furthermore, by using a composite score of CM, we adopted a more comprehensive approach that allowed us to take into account the functionally intertwined components of the cardiometabolic system.

## Conclusion

This research is the first to present evidence that evening chronotype is correlated with a range of cardiometabolic risk factors among Chinese young adults. Our results suggest that dysregulation of sleep duration by biological and social influences is an additional factor contributing to cardiovascular disease risk and highlight the potential of sleep and circadian rhythm interventions in preventive health care.

## Electronic supplementary material

Below is the link to the electronic supplementary material.

Supplementary Material 1

## Data Availability

The dataset for this study is kept in the School of Public health, at Anhui Medical University, China and may be available upon request (Xiaoyan Wu, xywu@ahmu.edu.cn).
