# Supplementary material for: Prospective study of the association between chronotype and cardiometabolic risk among Chinese young adults
Source: BMC Public Health. 2023 Oct 11;23:1966. doi: 10.1186/s12889-023-16902-2 (PMC10566129; doi:10.1186/s12889-023-16902-2)
Supplement: Supplementary file 1 — Supplementary Material 1 [file 12889_2023_16902_MOESM1_ESM.docx]

**Table S1** Pearson correlations between chronotype and cardiometabolic measures

| Variable | 1 | 2 | 3 | 4 | 5 | 6 | 7 | 8 | 9 | 10 | 11 |
| --- | --- | --- | --- | --- | --- | --- | --- | --- | --- | --- | --- |
| 1.MEQ-5 score | 1 |  |  |  |  |  |  |  |  |  |  |
| 2.TC | -0.012 | 1 |  |  |  |  |  |  |  |  |  |
| 3.TG | -0.050 | 0.382^**^ | 1 |  |  |  |  |  |  |  |  |
| 4.HDL-C | 0.014 | 0.242^**^ | -0.274^**^ | 1 |  |  |  |  |  |  |  |
| 5.LDL-C | 0.011 | 0.811^**^ | 0.312^**^ | -0.154^**^ | 1 |  |  |  |  |  |  |
| 6.GLU | -0.022 | -0.099^**^ | -0.009 | 0.051 | -0.172^**^ | 1 |  |  |  |  |  |
| 7.INS | -0.127^**^ | 0.264^**^ | 0.382^**^ | -0.241^**^ | 0.232^**^ | 0.087^*^ | 1 |  |  |  |  |
| 8.HOMA-IR | -0.126^**^ | 0.246^**^ | 0.371^**^ | -0.221^**^ | 0.204^**^ | 0.197^**^ | 0.990^**^ | 1 |  |  |  |
| 9.hs-CRP | 0.045 | 0.021 | 0.028 | -0.054 | 0.020 | -0.011 | 0.087^*^ | 0.089^*^ | 1 |  |  |
| 10.MAP | -0.013 | 0.061 | 0.084^*^ | -0.066 | 0.040 | 0.155^**^ | 0.122^**^ | 0.141^**^ | 0.087^*^ | 1 |  |
| 11.CM-risk score | -0.102^**^ | 0.196^**^ | 0.618^**^ | -0.574^**^ | 0.286^**^ | 0.085^*^ | 0.735^**^ | 0.730^**^ | 0.114^**^ | 0.467^**^ | 1 |

^*^ *P value* < 0.05, ^**^ *P value* < 0.001.

**Table S2** Pearson correlations between chronotype and 2 years later cardiometabolic measures

| Variable | 1 | 2 | 3 | 4 | 5 | 6 | 7 | 8 | 9 | 10 | 11 |
| --- | --- | --- | --- | --- | --- | --- | --- | --- | --- | --- | --- |
| 1.MEQ-5 score | 1 |  |  |  |  |  |  |  |  |  |  |
| 2.TC | 0.073 | 1 |  |  |  |  |  |  |  |  |  |
| 3.TG | -0.071 | 0.316^**^ | 1 |  |  |  |  |  |  |  |  |
| 4.HDL-C | -0.002 | 0.256^**^ | -0.095 | 1 |  |  |  |  |  |  |  |
| 5.LDL-C | 0.012 | 0.705^**^ | 0.277^**^ | -0.414^**^ | 1 |  |  |  |  |  |  |
| 6.GLU | -0.104 | -0.128^*^ | 0.069 | -0.046 | -0.021 | 1 |  |  |  |  |  |
| 7.INS | -0.161^**^ | -0.012 | 0.069 | -0.176^**^ | 0.164^**^ | 0.348^**^ | 1 |  |  |  |  |
| 8.HOMA-IR | -0.158^**^ | -0.015 | 0.077 | -0.177^**^ | 0.163^**^ | 0.424^**^ | 0.993^**^ | 1 |  |  |  |
| 9.hs-CRP | -0.184 | -0.154 | 0.139 | -0.314^**^ | -0.022 | -0.007 | 0.110 | 0.115 | 1 |  |  |
| 10.MAP | -0.135^*^ | 0.029 | 0.270^**^ | 0.077 | 0.001 | 0.212^**^ | 0.096 | 0.111 | 0.174 | 1 |  |
| 11.CM-risk score | -0.179^**^ | 0.020 | 0.474^**^ | -0.413^**^ | 0.302^**^ | 0.366^**^ | 0.667^**^ | 0.681^**^ | 0.139 | 0.498^**^ | 1 |

^*^ *P value* < 0.05, ^**^ *P value* < 0.001.
